# Neuropathological diagnosis of vascular cognitive impairment and vascular dementia with implications for Alzheimer’s disease

**DOI:** 10.1007/s00401-016-1571-z

**Published:** 2016-04-09

**Authors:** Raj N. Kalaria

**Affiliations:** Institute of Neuroscience, Newcastle University, Campus for Ageing and Vitality, Newcastle upon Tyne, NE4 5PL UK

**Keywords:** Alzheimer’s disease, Cerebral amyloid angiopathy, Cerebrovascular degeneration, Dementia, Neuropathology, Small vessel disease, Vascular dementia

## Abstract

Vascular dementia (VaD) is recognised as a neurocognitive disorder, which is explained by numerous vascular causes in the general absence of other pathologies. The heterogeneity of cerebrovascular disease makes it challenging to elucidate the neuropathological substrates and mechanisms of VaD as well as vascular cognitive impairment (VCI). Consensus and accurate diagnosis of VaD relies on wide-ranging clinical, neuropsychometric and neuroimaging measures with subsequent pathological confirmation. Pathological diagnosis of suspected clinical VaD requires adequate postmortem brain sampling and rigorous assessment methods to identify important substrates. Factors that define the subtypes of VaD include the nature and extent of vascular pathologies, degree of involvement of extra and intracranial vessels and the anatomical location of tissue changes. Atherosclerotic and cardioembolic diseases appear the most common substrates of vascular brain injury or infarction. Small vessel disease characterised by arteriolosclerosis and lacunar infarcts also causes cortical and subcortical microinfarcts, which appear to be the most robust substrates of cognitive impairment. Diffuse WM changes with loss of myelin and axonal abnormalities are common to almost all subtypes of VaD. Medial temporal lobe and hippocampal atrophy accompanied by variable hippocampal sclerosis are also features of VaD as they are of Alzheimer’s disease. Recent observations suggest that there is a vascular basis for neuronal atrophy in both the temporal and frontal lobes in VaD that is entirely independent of any Alzheimer pathology. Further knowledge on specific neuronal and dendro-synaptic changes in key regions resulting in executive dysfunction and other cognitive deficits, which define VCI and VaD, needs to be gathered. Hereditary arteriopathies such as cerebral autosomal dominant arteriopathy with subcortical infarcts and leukoencephalopathy or CADASIL have provided insights into the mechanisms of dementia associated with cerebral small vessel disease. Greater understanding of the neurochemical and molecular investigations is needed to better define microvascular disease and vascular substrates of dementia. The investigation of relevant animal models would be valuable in exploring the pathogenesis as well as prevention of the vascular causes of cognitive impairment.

## Introduction

Cerebrovascular disease (CVD) is the second most common cause of age-related cognitive impairment and dementia, which is widely recognised as vascular dementia (VaD). VaD culminates from global or localised effects of vascular disease, which incurs stroke injury and other tissue perfusion changes. VaD is characterised as a neurocognitive disorder, but also incorporates behavioural symptoms, locomotor abnormalities and autonomic dysfunction. Vascular cognitive impairment (VCI) results from all causes of CVD including cardiovascular that lead to early and late plus severe forms of dementia syndromes. Within CVD, the most common vascular contributor to dementia is likely cerebral small vessel disease (SVD), which describes a range of clinical, neuroimaging and pathological features. SVD has taken precedence as a radiological concept, but refers to an intracranial disorder that encompasses pathological changes within and at the surfaces of brain microvessels including perforating arteries and arterioles, capillaries and venules. SVD involves tissue injury in both the cortical and subcortical grey and white matter (WM). SVD, however, may often coexist with atherosclerosis involving large extracranial vessels and embolic disease [103].

In this article, I review the brief history of our current understanding of VaD, various criteria incorporating clinical, neuropsychological and pathological features that have been proposed over the years and key vascular lesions and tissue changes, which contribute to dementia. I convey some opinions about brain sampling and consider some of the rarer causes of VCI and VaD and how these can be investigated. It is clear that despite the strong and unambiguous evidence that vascular factors and vascular disease contribute to the global burden of brain disease, dementia prognosis and research has mostly focused on Alzheimer’s disease (AD). Vascular causes of dementia and their contribution to neurodegenerative processes have not been widely emphasised.

## Historical aspects and nosology

One could begin with Thomas Willis and apoplexy, but the concept that gradual strangulation of the brain causes cognitive and behavioural deficits was distinguished just over 100 years ago [18]. Both Alzheimer and Kraeplin had reasoned that old age-associated progressive hardening of the arteries lead to arteriosclerotic dementia. The label arteriosclerotic dementia attributed to cerebral softening with loss of relatively large volume (50–100 mL) of brain tissue was used in hospital records as late as the 1960s [165]. The diagnosis of arteriosclerotic dementia often superseded that of AD, which became to be frequently diagnosed in the late 1970s on whether it is a form of pre-senile or senile dementia. Unlike arteriosclerotic dementia, the current formulation of VaD has transformed as a distinct condition over the past 25 years. VaD or cerebrovascular dementia implies a clinically diagnosed dementia syndrome comprising subtypes with both ischemic and haemorrhagic aetiologies [142]. As AD became more commonly recognised, VaD was often similarly characterised as a primary memory-associated dementia but involving vascular causes.

Otto Binswanger could probably be acknowledged to have conveyed the notion of the existence of subclasses of VaD. He described subcortical arteriosclerotic encephalopathy or a type of SVD-related dementia [18]. This was described after pathological verification of cerebral WM disorder in a group of patients with hypertensive disease. Further descriptions of distinct pathological changes in cerebral vessels were another step forward towards classification of subtypes. In 1937, W Schultz had described *drusige entrartung* or congophilic amyloid angiopathy in some patients. More recently, C. Miller Fisher recognised for his profound proposal indicated that cerebrovascular dementia is a matter of both large and small strokes and provided clear accounts of lacunar syndromes [56]. Multiple small infarcts in association with hypertension (*état lacunaire*) are the commonest pathological changes linked to VaD. It is characterised by abrupt episodes, which lead to weakness, slowness, dysarthria, dysphagia, small-stepped gait, brisk reflexes and extensor plantar responses. All these signs are largely present by the time mental deterioration occurs [73]. The recognition of subtypes of clinical VaD was clearly an important step towards current pathological classifications based on vascular aetiology. It was subsequently recognised that multi-infarct dementia predominantly results from cortical infarcts attributed to large vessel disease, whereas dementia associated with subcortical ischemic lesions or Binswanger’s disease involving subcortical structures and the WM results from changes in intracranial small vessels (Table 1).

**Table 1:** Common and uncommon causes of stroke pathophysiology associated with cognitive impairment or dementia

Primary or secondary vascular disorder(s)^a^	Common conditions	Vascular distribution	Predominant tissue changes	Form(s) of VaD/major VCD^**b**^
Atherosclerotic disease	Carotid and cardiac atherosclerosis	Aorta, carotid, intracranial- MCA branches	Cortical and territorial infarcts; WML	Large vessel dementia or multi-infarct dementia
		Aorta, coronary	Infarcts, laminar necrosis, rarefaction	Hypoperfusive dementia
Embolic disease	Cardio or carotid embolism	Intracranial arteries, MCA	Large and small infarcts	Multi-infarct dementia
Arteriolosclerosis	Sporadic small vessel disease	Perforating and penetrating arteries, lenticulostriate arteries	Cortical infarcts, lacunar infarcts/lacunes, microinfarcts, WML	Small vessel dementia; subcortical ischaemic vascular dementia; strategic infarct dementia
	Hypertensive vasculopathy			Hypertensive encephalopathy with impairment; strategic infarct dementia
Non-atherosclerotic non-inflammatory vasculopathies	Arterial dissections (carotid, vertebral and intracranial), fibromuscular dysplasia, dolichoectatic basilar artery, large artery kinking and coiling, radiation induced angiopathy, moyamoya disease	Vertebral, basilar, branches of MCA, mural haematoma perforating artery; SVD	No pattern of brain infarctions: haemodynamic, thromboembolic, or due to occlusion of a perforating artery. Subarachnoid haemorrhage; lacunar infarcts, PVS	Vascular cognitive impairment
	Aneurysms—saccular, berry, fusiform, cerebral	Circle of Willis, proximal branches of MCA, PCA	Haemorraghic infarcts, herniation	Haemorrhagic dementia
	Vascular malformations: cavernous hemiangioma, arteriovenous, capillary	Cortical lobes	Rarefaction, WML	Vascular cognitive impairment
	Cerebral venous thrombosis	Venous sinus, periventricular veins	Subcortical infarcts (thalamus), lobar haemorrhages	
Amyloid angiopathies	Hereditary CAAs (amyloid β, prion protein, cystatin C, transthyretin, gelsolin)	Leptomeninges, intracerebral arteries	Cortical microinfarcts, lacunar infarcts, WML	Vascular cognitive impairment, dementia
Monogenic stroke disorders	CADASIL, CARASIL, retinal vasculopathy with cerebral leukodystrophies (RVCLs), Moyamoya disease, hereditary angiopathy, nephropathy, aneurysm and muscle cramps (HANAC)	Leptomeningeal arteries, intracerebral subcortical arteries	Lacunar infarcts/lacunes, microinfarcts, WML	Vascular cognitive impairment, dementia
Monogenic disorders involving stroke	Fabry disease, familial hemiplegic migraine, hereditary haemorrhagic telangiectasia, vascular Ehlers–Danlos syndrome, Marfan syndrome, psuedoxanthoma elasticum, arterial tortuosity syndrome, Loeys–Dietz syndrome, polycystic kidney disease; neurofibromatosis type 1 (von Ricklinghausen disease), Carney syndrome (facial lentiginosis and myxoma)	Branching arteries	Cortical and subcortical infarcts, haemorraghic infarcts	Vascular cognitive impairment, dementia
Metabolic disorders	Mitochondrial disorders (MELAS, MERRF, Leigh’s disease, MIRAS), Menkes disease, homocystinuria, Tangier’s disease	Intracerebral small arteries, territorial arteries	Cortical and subcortical stroke-like lesions, microcystic cavitation, cortical petechial haemorrhages, gliosis, WML	Vascular cognitive impairment
Haematological disorders	Paraproteinaemia, coagulopathies (antiphospholipid antibodies, SLE, nephrotic syndrome, Sneddon syndrome, deficiencies in clotting cascade factors, e.g. protein S, C, Z, antithrombin III, plasminogen)	Large and intracerebral arteries	Cortical and subcortical infarcts, ICH and subarachnoid haemorrhages	Vascular cognitive impairment
Vasospastic disorders	Subarachnoid haemorrhage, migraine-related strokes, paroxysmal hypertension, drug-induced vasoconstriction	Intracranial arteries, MCA	Cortical and subcortical small infarcts	Vascular cognitive impairment

Data summarised from several source references [28, 52, 53, 88]. Several disorders may also occur with other co-morbidities such as coronary artery disease, congestive heart failure, hypertension, diabetes, hyperlipidaemia, hypercoagulability, renal disease, atrial fibrillation and valvular heart disease

*CAA* cerebral amyloid angiopathy, *CADASIL* cerebral autosomal dominant arteriopathy with subcortical infarcts and leukoencephalopathy, *CARASIL* cerebral autosomal recessive arteriopathy with subcortical infarcts and leukoencephalopathy, *ICH* intracerebral haemorrhage, *MCA* middle cerebral artery, *MELAS* mitochondrial myopathy, encephalopathy, lactic acidosis and stroke-like episodes, *MERRF* myoclonic epilepsy with ragged red fibres, *MIRAS* mitochondrial recessive ataxic syndrome, *PCA* posterior cerebral artery, *PVS* perivascular spaces, *SLE* systemic lupus erythematosus, *SVD* small vessel disease, *VaD* vascular dementia, *VCD* vascular cognitive disorder, *WML* white matter lesion

^a^Other miscellaneous causes of stroke including mechanical, invention induced or rare genetic syndromes such as trauma, iatrogenic, decompression sickness, air or fat embolism and transplantation and Werner’s syndrome can lead to cognitive impairment

^b^VCI determined when two or more cognitive domains are affected per minimal harmonisation guidelines or minor VCD [72, 146]

## The continuum of VCI and vascular cognitive disorder

VCI came into existence to empower a single label for all conditions in any cognitive domain that has a vascular origin or impaired brain perfusion [118]. While useful, it is challenging to consistently correlate the degree of pathological changes with the degree of impaired cognition in the continuum of VCI [65, 72, 118]. The description vascular cognitive disorder [145] also incorporates a continuum comprising cognitive disorders of vascular aetiology with diverse pathologies and clinical manifestations. Therefore, in the most recent diagnostic and statistical manual of mental disorders (DSM) or DSM-V criteria and guidelines, the categories of mild and major vascular cognitive disorders were introduced [8]. Vascular cognitive disorder indicated a global diagnostic category, restricting the term VCI to patients whose cognitive impairment fell short of dementia [142]. The major neurocognitive disorder classification, meant to describe frank dementia as a substitute for VaD, appears to fit better with patients and more adapted to neurodegenerative cognitive disorders for which memory impairment is not predominant, but comprises substantial frontal lobe pathology [146].

Cognitive impairment or dementia following stroke is recognised to be relatively common [102, 129]. Incident dementia after stroke or post-stroke dementia (PSD) has become better defined in recent years. PSD may develop within 3 months or after a stabilisation period of a year or longer after stroke injury [4, 16, 133]. However, PSD can have a complex aetiology with varying combinations of large and SVD as well as non-vascular pathology. Stroke injury or CVD may unmask other preexisting disease processes such as AD. It has been recently demonstrated that at least 75 % of PSD cases fulfilling relevant clinical guidelines for VCI are pathologically confirmed as VaD with little or no AD pathology [4]. Thus, most of PSD is VaD.

## Clinical information on vascular causes of dementia

Review of the medical records of a patient who has died with CVD provides insight into the nature of clinical progression and identifies anatomical regions linked to any patterns of changes in cognition or behaviour. It also assists in planning extra histological sampling in addition to the standard brain cutting and sampling procedures (Table 2). Strategies for the staged examination of the postmortem brain in suspected dementia have evolved over time with the increasing use of immunohistochemical and molecular tools for diagnosis. This has led to an expanding range of diagnostic categories (Table 1). In CVD cases, diagnostic imaging may have been performed that will also be useful for the diagnosis. However, clinical information plays an important role in the formulation of a clinicopathological summary. Thus, a number of questions should be considered: (1) How was the diagnosis of dementia made? (2) Was the assessment been made by a clinician experienced in dementia? (3) Have causes of secondary dementia been excluded? (4) Has there been longitudinal assessment of the patient with application of bedside tests of cognitive function? It is common for a diagnosis of dementia to be applied to an elderly subject who has delirium or is cognitively impaired because of an acute problem and is therefore best classed as having an acute confusional state? Depression may also lead to poor global performance and is a recognised cause of pseudo-dementia. Another question relates to the domains of cognition affected first. At the end stage of disease, it can be clinically difficult to discriminate between different diseases. The early clinical features obtained from medical records often give important clues to the subsequent pathological diagnosis for which the pathologist attempts to distinguish between the various clinico-anatomic syndromes [88]. Further specific questions concern neurological features associated with the decline in cognitive function that can be attributed to the cause of dementia.

**Table 2:** Pathological lesions in CVD for neuropathology reporting

Key variables for pathological diagnosis
Ischaemic or haemorrhagic infarct(s)
Is the haemorrhagic lesion(s) a major component?
Gross pathological features^a^
Atherosclerosis (basal, peripheral or meningeal), large infarcts, haemorrhage, herniation, malformations, atrophy
Microscopic vascular changes^b^
Microvascular disease (sporadic, hyertensive)
Microvascular disease (e.g. CAA)
Other microangiopathies
Small vessel disease changes: lipohyalinosis; fibroid necrosis, hyalinisation, collagenosis
Perivascular dilatation
Parenchymal changes^b^
Location: cortex, WM, basal ganglia, brainstem (pontine), cerebellum
Circulation involved: arterial territories—anterior, middle or posterior
Laterality: right or left anterior and posterior
Sizes/number of infarcts = dimension: 0–4, 5–15, 16–30, 31 > 50 mm
Microinfarction; <5 mm determined as small or microinfarcts
Lacunes and lacunar infarcts: *etat lacunaire *and* etat crible *(grey and WM)
Leukoencephalopathy (WMD): anterior vs. posterior; periventricular vs deep WM
Rarefaction/incomplete or subinfarctive ischemic injury
Degree of perivascular and parenchymal gliosis: mild, moderate or severe
Hippocampal sclerosis: mild, moderate and severe
Alzheimer pathology (NFT, neuritic plaque staging). >stage III = mixed AD and VaD

*CAA* cerebral amyloid angiopathy, *CVD *cerebrovascular disease, *NFT* neurofibrillary tangles, *WM* white matter, *WMD *white matter disease

^a^ *Gross examination* The protocol for examination of brains from CVD subjects is essentially similar to that for any other disease. The routine includes looking for sites and volumes of haemorrhages, herniation, malformations, swelling or oedema and atrophy. Any extradural, subdural or subarachnoid haemorrhage(s) that has occurred should be noted. There may be signs of ruptured aneurysms, cortical lacerations, burst intracranial haemorrhage and leakage of intraventricular haemorrhage through the cerebellar foramina. The basal cerebral arteries and vertebro-basilar arteries and the main branches can be checked for the degrees of atheroma and the presence of thrombosis. Open branch points, for example, at the trifurcation of the internal carotid and middle cerebral artery are common sites for emboli. Vascular abnormalities may include aneurysms, clips and endovascular coils and malformations. The leptomeninges should be assessed for thickness and translucency, which may be altered much with age

^b^For reporting purposes, each of the above features can be scored numerically to provide a summary [72]. For example, 0 is absent and 1 means present. Less frequent lesions including watershed infarcts and laminar necrosis. Increasing numerical value may also be assigned to the infarcts

## Neuropsychometric correlates of VCI and VaD

Upon evaluation of clinical information including history, timing of event, neuropsychometry and neuroimaging of the DSM criteria are mostly widely applied to define the presence of dementia. In the DSM-IV and earlier versions of DSM criteria, diagnosis of dementia placed emphasis on memory loss as a core feature. However, many patients with VaD will not necessarily have profound memory deficits, particularly in the early stages. They predominantly develop a frontal dysexecutive syndrome [40]. This shortfall has been overcome in recently proposed guidelines for the diagnosis of minor and major vascular cognitive disorder, which concentrates on speed of information processing, complex attention and frontal-executive functioning [146]. Another advancement in this context is the use of the Montreal Cognitive Assessment (MoCA) as a preferred first cognitive screening instrument to challenge the well-established Mini-Mental State Exam (MMSE). Both the full and short versions of the MoCA appear to have excellent diagnostic accuracy in discriminating VaD patients in terms of sensitivity and specificity against the MMSE [58]. To define various cognitive domains for assessment of executive dysfunction including features such as processing speed, attention and reaction time, different centres use variations of established neuropsychometric batteries and tests such as the Automated Geriatric Examination for Computer-Assisted Taxonomy (AGECAT), Cambridge Cognitive Examination (CAMCOG), Cambridge Examination for Mental Disorders (CAMDEX), Cognitive Abilities Screening Instrument (CASI) and Mattis Dementia Rating Scale (MDRS), which are most often biased for AD (Table 3).

**Table 3:** How was the burden of vascular pathology assessed in ageing and dementia studies?

Study [ref.]	Type of sample	Sample size	Brain regions	Vascular lesions (VLs)/vascular brain injury (VBI)	Scoring system	Dementia criteria and cognitive tests
CFAS [29]	Ageing	209	12 standard sections	Infarct, small vessel disease (arteriolosclerosis), CAA, periarteriolar myelin attenuation with gliosis, leukoencephalopathy	CERAD, 0–3 (none, mild moderate, severe)	MMSE, AGECAT
Rochester Epidemiology Project [95]	Ageing	89	Ten standard sections	Large infarcts, lacunes, (assessed bilaterally in grey and white matter), leukoencephalopathy	% frequency	DSM-IV, NINDS-ARIEN, ADDTC, ICD-10
Geriatric and Psychiatric Hospitals, Geneva [63]	Mixed	156	Six large coronal section	Lacunes, cortical microinfarcts diffuse, CAA, focal gliosis, periventricular and deep WM demyelination	Vascular score (CMI and thalamic and basal ganglia lacune score), 0–20	CDR (0–3)
Rush Memory and Ageing Project [148]	Ageing, AD	148	8–9 standard sections	Macroscopic and microscopic infarcts (acute, subacute, chronic).	% infarcts	MMMSE, Complex Ideational Material, NINDS-ADRDA
Bronx Ageing Study, Einstein Ageing Study, AE Nursing Home study [158]	AD, VaD, Mixed	190	15 standard sections	Large infarcts, lacunes, leukoencephalopathy	Vascular lesion score, 0–6	DSM-IIIR, DSM-IV, NINDS-ADRDA, ADDTC
HAAS [100, 179]	Ageing, AD	436	Eight standard sections	Large infarcts, lacunes, microinfarcts, leukoencephalopathy (myelin loss with gliosis), haemorrhages	% infarcts, median no. 25th and 75th percentiles	CASI, CDR (0–3)
Adult Changes in Thought study [105, 157]	Ageing	219	10 standard sections	Macroinfarcts (<1 or >1 cm), microinfarcts, leukoencephalopathy (myelin loss), haemorrhages	Frequency of lesions	DSM-IIIR, DSIM-IV, NIA-Reagan Institute
NACC [164]	AD	4629	12 standard sections	Large infarcts, lacunes, microinfarcts, leukoencephalopathy (myelin loss), haemorrhages, atherosclerosis (CW), arteriolosclerosis, CAA	% VLs, plus 0–3 (none, mild moderate, severe)	DSM-IIIR, DSIM-IV, NIA-Reagan Institute
Oxford [48]	CVD, VaD	61	Six standard sections	Large infarcts, lacunes, microinfarcts, CAA, cribriform change, perivascular spacing and arteriolosclerosis (SVD), atherosclerosis (CW)	Infarcts graded (no, single, multiple); SVD and atheromas, 0–3	MMSE, CAMDEX, Kew test
OPTIMA [155]	Ageing, AD	70	Four standard sections	Small infarcts, microinfarcts, leukoencephalopathy	SVD 0–3 (none, mild, moderate, severe)	MMSE, CAMCOG
CogFAST [39]	AD, VaD, Mixed, DLB	135; 226	Four large coronal sections	Large and small infarcts, lacunes, microinfarcts, arteriolosclerosis, CAA, perivascular hemosiderin leakage, perivascular spaces, leukoencephalopathy (myelin loss)	Vascular score, 0–20	DSM-IIR, DSM-IV, MMSE, CAMCOG

Essential data taken from several references as shown

*AD* Alzheimer’s disease, *ADDTC* Alzheimer’s Disease Diagnostic and Treatment Centers, *AGECAT* automated geriatric examination for computer-assisted taxonomy, *CAA* cerebral amyloid angiopathy, *CASI* Cognitive Abilities Screening Instrument, *CAMCOG* Cambridge Cognitive Examination, *CAMDEX* Cambridge Examination for Mental Disorders, *CDR* Clinical Dementia Rating, *CFAS *Cognitive Function in Ageing Study, *CVD* cerebrovascular disease, *DLB* dementia with Lewy bodies, *HAAS *Honolulu Asia-Aging Study, *Mixed* mixed dementia both AD and VaD, *MMSE* Mini-Mental State Examination, *NACC* National Alzheimer's Coordinating Centre, *VaD* vascular dementia, *VLs* vascular lesions

## Towards the diagnostic criteria for VaD

In the past, several proposals were made to better define the diagnostic criteria for VaD [182, 183]. These have variable specificities and sensitivities and are not interchangeable with substantial misclassification of dementias [35, 62, 135]. The inclusion of deficits in certain cognitive domains such as memory, which is primary to AD, concurs with the relatively low sensitivity (0.20), but high specificity (0.93) for probable VaD apparent in clinicopathological validation studies [62]. In earlier studies, the Hachinski Ischaemic Scale used was used to indicate the presence of multi-infarct dementia in demented patients who scored ≥7 out of 10. Subsequent specific developments included the Alzheimer’s Disease Diagnostic and Treatment Centers (ADDTC) criteria for ischaemic VaD [33], the National Institute for Neurological Disorders and Stroke-Association Internationale pour la Recherché et l’Enseignement en Neurosciences (NINDS-AIREN) criteria for VaD [144] and the International Classification of Diseases (ICD-10) criteria for VaD. The ADDTC followed by the NINDS-AIREN criteria for possible (ischaemic) VaD achieves the best balance of sensitivity and specificity with reasonable agreement with DSM-IV criteria for possible VaD. However, none of the criteria including the ADDTC, NINDS-AIREN and ICD-10 consistently revealed high sensitivity for probable VaD. Despite the deficiencies, the NINDS-AIREN criteria are still most widely used, particularly in research settings. The NINDS-AIREN criteria emphasise the heterogeneity of VaD syndromes and pathological subtypes (e.g. ischaemic and haemorrhagic strokes, cerebral hypoxic–ischaemic events, WM changes) [144]. The three cardinal features of VaD that harmonise with NINDS-AIREN criteria for the clinical diagnosis of probable VaD include (1) acute onset of dementia, demonstrated by impairment of memory and two other cognitive domains, such as orientation, praxis, or executive dysfunction, (2) relevant neuroimaging evidence of cerebrovascular lesions and (3) evidence for a temporal relation between stroke and cognitive loss [142]. Although neuroimaging evidence of vascular lesions is required for a diagnosis of probable VaD, the NINDS-AIREN criteria do not distinguish between subjects with and without dementia in the context of CVD [11]. The diagnosis of ‘definite’ VaD requires histopathological evidence of CVD (Table 2), an absence of neurofibrillary tangles and neuritic plaques exceeding those expected for age and an absence of other conditions associated with dementia [90].

Despite the wide use of NINDS-AIREN and DSM-IV criteria, postmortem examination is not performed in general. However, when they do occur, inaccuracy of clinically diagnosed VaD is often revealed. Invariably, autopsy findings reveal subjects with AD type of pathological changes [45, 80, 87]. For example, a US study [117] reported that 87 % of the patients enrolled in a prospective series to examine VaD in a dementia clinic setting were found to have AD either alone (58 %) or in combination with CVD (42 %). All of the patients with signs of CVD were also found to have some concomitant neurodegenerative disease. Similarly, another study indicated that large numbers of ‘pure’ VaD cases without co-existing neuropathological evidence of AD are uncommon [80]. This means that the current clinical diagnostic criteria are useful to detect pathology, but not necessarily “pure” pathology [79, 95]. There are currently no widely validated criteria for either VCI or vascular cognitive disorder [63, 72, 146]. Unbiased criteria encompassing relevant cognitive domains for VCI still need to be widely evaluated [39, 65, 72]. However, as with AD, definitive diagnosis of VaD is made at autopsy, but appropriate sampling and essential neuropathological examination are necessary to rule out significant other pathological changes associated with different causes of cognitive impairment [72].

Several factors account for the difficulty in deriving an accurate diagnosis of VaD. These include sampling bias, inadequate sample size and absence of pathological verification in many clinical studies; the use of non-standard or difficult-to-compare assessment instruments for clinical, neuropsychological, neuroimaging and neuropathological evaluation [72, 127]; and, equally important, disagreement over interpretation of data. More sensitive neuroimaging modalities have increased antemortem recognition of vascular changes in dementia patients, but these have also become harder to interpret, by revealing similar lesions in non-demented individuals. As discussed above, accurate diagnosis is also not straightforward given the heterogeneity of vascular lesions and the inherent issues with standardisation, especially when assessing mixed pathologies [63]. Depending on the inclinations of the observer, cases of AD with coexistent vascular lesions such as infarcts may be classified variously as VaD, or AD with coexistent vascular pathology, or mixed dementia [55, 149]. To derive more accurate prevalence or incidence estimates and pathological diagnosis, uniformity in protocols and appropriate brain sampling at autopsy across different centres are necessary [3, 39, 63, 72, 127, 158].

## Clinicopathological correlation in VaD: past and present

Although diagnostic criteria for the neuropathological validation of VaD are lacking, neuroimaging and clinicopathological studies have clearly indicated that the threshold for VaD depends on the extent of cerebral damage. A combination of factors including origin, volume, location and number of lesions contribute to the development of dementia. Tomlinson and colleagues had previously determined that the total volume of infarcts in demented stroke patients was usually over 50 mL and in some cases greater than 100 mL, exceeding that in non-demented stroke patients [19, 165]. Subsequent clinicopathological studies reported that only 5 of 23 patients with a pathological diagnosis of VaD had more than 50 mL of infarcted tissue and 7 had less than 10 mL [45]. It is now clear that widespread small ischaemic lesions or multiple microinfarcts [178, 179] distributed throughout the CNS correlate better with dementia and are key predictors of cognitive impairment [86]. Location of lesions may also be more critical than total volume [41, 46]. For example, infarction in the left hemisphere disproportionately increases the risk of dementia [41, 64, 104, 134]. Bilateral infarcts with greater involvement of the dominant hemisphere also increase the risk of dementia after stroke [38, 45, 104].

Relatively few prospective studies have validated criteria for VaD. Previous criteria for Binswanger’s disease or cerebral SVD [17] proposed that the clinical diagnosis of dementia accompanied by neuroimaging evidence (CT or MRI) of bilateral abnormalities and at least two of three findings included evidence of (1) a vascular risk factor or systemic vascular disease, (2) focal cerebrovascular disease and (3) “subcortical” cerebral dysfunction described by gait disorder, parkinsonism, or incontinence. These criteria were validated in a prospective series of 184 patients with AD and showed that only 1.6 % were diagnostically misclassified when all three clinical criteria were met [17].

The Oxford Project to Investigate Memory and Ageing (OPTIMA) study has recently developed a simple, novel, image-matching scoring system [155] to relate the extent of SVD with cognitive function in a study of 70 cases with insufficient pathology to meet the criteria for the diagnosis of AD. The severity of SVD pathology was inversely related to cognitive scores and 43 % of the cases with high SVD scores were designated as being demented. To better define clinicopathological correlation in subtypes of VaD including SVD, a staging system related to the natural history of cerebrovascular pathology and an algorithm for the neuropathological quantification of the CVD burden in dementia have been proposed [39]. The staging system (I–VI) needs further evaluation against cognitive function scores to determine whether this system can be used in large-scale studies to understand the clinicopathological correlations.

**Fig. 1:**
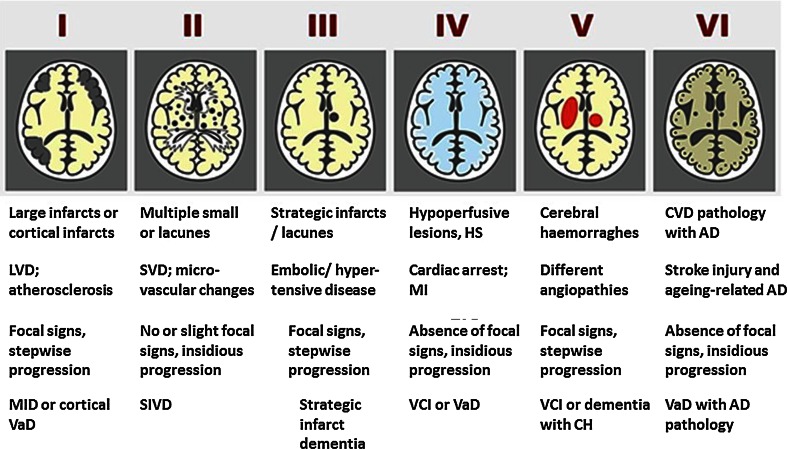
Schematic diagram of different cerebrovascular pathologies associated with dementia. The proposed Newcastle categorisation includes six subtypes [90]. In all the above, the age of the vascular lesion(s) should correspond with the time when the disease began. The post-stroke survivors are usually included in subtypes* I*–*III*. While these may not be different from other published subtypes [84], they are practical and simple to use. Cases with extensive WM disease in the absence of significant other features are included under SVD. *Subtype* I* may result from large vessel occlusion (atherothromboembolism), artery to artery embolism or cardioembolism. Subtype* II* usually involves descriptions of arteriosclerosis, lipohyalinosis and hypertensive, arteriosclerotic, amyloid or collagen angiopathy. Subtypes* I*,* II* and* V* may result from aneurysms, arterial dissections, arteriovenous malformations and various forms of arteritis (vasculitis). *AD* Alzheimer’s disease, *CH* cerebral haemorrhage, *CVD* cerebrovascular disease, *MI* myocardial infarction, *MID* multi-infarct dementia, *LVD* large vessel disease, *SIVD* subcortical ischaemic vascular dementia, *SVD* small vessel disease, *VCI* vascular cognitive impairment, *VaD* vascular dementia

Neuropathological diagnosis of VaD should be based on the absence of a primary neurodegenerative disease known to cause dementia and the presence of cerebrovascular pathology that defines one or more of the VaD subtypes (Table 1). These would also include dementia among post-stroke survivors who fulfill the NINDS-AIREN criteria [144] for probable VaD. Stroke survivors with mild cognitive impairment or VCI [118] may also have sufficient pathology for neuropathological diagnosis of VaD [4]. A proposal for the neuropathological diagnostic evaluation of VaD was previously published by the Newcastle investigators (Fig. 1). According to these criteria, there are two neuropathological diagnostic groups: probable VaD is based on the exclusion of a primary neurodegenerative disease known to cause dementia plus the presence of cerebrovascular pathology that defines one or more of the VaD subtypes. Possible VaD is designated when the brain contains vascular pathology that does not fulfill the criteria for one of the subtypes, but where no other explanation for dementia is found. Post-stroke survivors are often classed as subtypes I–III. Cases with extensive WM disease in the absence of other significant pathologies are included under SVD.

Assessing the neuropathological substrates of VaD involves systematic assessment of parenchymal lesions, including microinfarcts and haemorrhages and the vascular abnormalities that may have caused them to relate to the progression of impairment [39, 90, 110, 155, 158]. In addition, systemic factors (e.g. hypotension, hypoglycaemia) may cause brain or neuronal lesions in the absence of severe vascular disease and should be taken into account when attributing causes to VaD. As discussed above, parenchymal abnormalities of neurodegenerative type may be present that are not obviously associated with either vascular disease or systemic factors, i.e. Alzheimer type or hippocampal lesions.

## Frequency of pathologically diagnosed VaD

Confirmation of VaD diagnosis is definitive at autopsy derived from appropriate sampling of both cerebral hemispheres and neuropathological examination [72] to rule out significant pathological changes associated with other dementias. The prevalence of early-onset dementia VaD (<65 years old) ranges from 3.1 to 44 % in various clinic and population-based studies across the world [172]. However, these values may not reflect the true prevalence and incidence rates of VaD due to inconsistencies in diagnostic criteria, sampling methods and subject or country demographics and variation in morbidity and mortality trends. When a range of clinical criteria was applied to sample sizes of 59–1929, autopsy studies showed that pathologically diagnosed VaD ranges widely from as low as 0.03 % to as high as 58 % with an overall mean estimate of 17 % [84]. In Western countries, the estimated rates of pathologically diagnosed VaD as defined by various criteria lie between 8 and 15 %. In studies where diagnosis was restricted to the currently used NINDS-AIREN criteria [144], the frequencies are reported to be ~7 %. Taking the above estimates into consideration, the worldwide frequency of VaD in autopsy-verified cases is calculated to 10–15 %, being marginally less than when clinical criteria alone are used [13, 95]. In Japan, the incidence of autopsy-verified VaD was previously 35 % [150] and later reported to be 22 % [1]. Population-based cohorts should provide the best estimates for pathology-verified VaD. However, there are only few such studies and they all show that microvascular lesions occur more frequently than neurodegenerative lesions in elderly community-dwelling subjects with dementia [29, 148, 157, 181].

## Sampling and investigation of the brain

Some form of CVD is common among the assortment of all routine autopsies. Stroke is the most frequent CVD disorder with more than 200 causes. Stroke-related injury may comprise microscopic lesions such as microinfarcts and microhaemorrhages to large cortical infarcts and lobar haemorrhages (Table 1). Recent advances in neuroimaging and systematic neuropathological examination have enabled better definitions of clinically diagnosed CVD, which causes cognitive impairment [72]. The pathological diagnosis of VaD or VCI, however, requires the systematic evaluation of potentially relevant clinical or phenotypic features with particular attention to the timing of events [88]. It is difficult to define which neuropathological changes and to what degree these contribute to dementia because of the heterogeneous localisation of lesions and the co-existence of other pathologies including neurodegenerative changes such as those in AD. More than one factor may contribute to the overall impairment and the VaD phenotype (Table 1). These include the origin and type of vascular occlusion, presence of haemorrhage, distribution of arterial territories and the size of vessels involved. Thus, many brain regions including the territories of the anterior, posterior and middle cerebral arteries, the angular gyrus, caudate and medial thalamus in the dominant hemisphere, the amygdala and hippocampus, as well as the hippocampus have been implicated in VaD. Factors that define pathology in subtypes of VaD include multiplicity, size, anatomical location, laterality and age of the lesions besides genetic influences and previous existence of systemic vascular disease. Subcortical ischaemic VaD is likely the most significant subtype of VaD [142] and smaller subcortical lesions seem to be key players (Table 2).

**Fig. 2:**
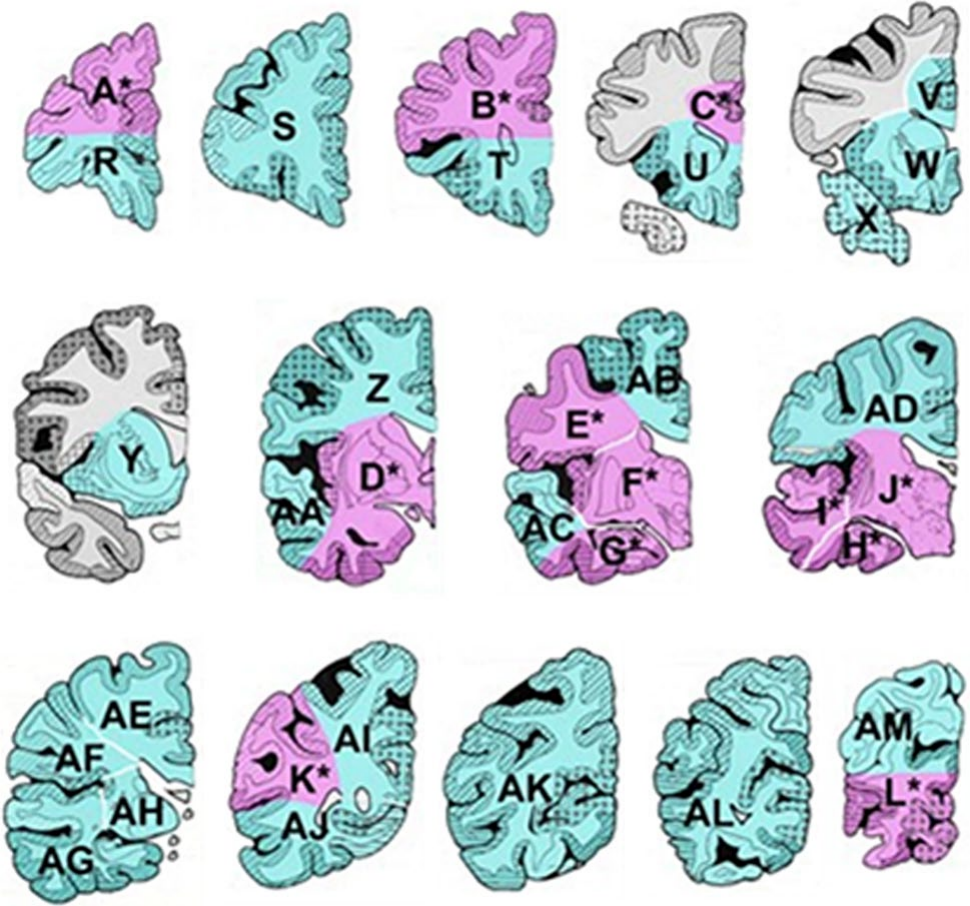
Sampling of postmortem brain tissue for assessing vascular pathology. Coronal blocks from one hemisphere (rostral to caudal) of the cerebrum for an ‘ideal’ sample for neuropathological assessment. In Newcastle, large sections are taken as indicated by the *pink* and *green blocks* identified by the *letters*. A minimum sample constituting four to six large blocks including S, Y/W, F/J, G/H, AB/AD and AL can be reliably used to determine the burden of vascular pathology [39]

Gross external examination of the brain at autopsy is extremely useful for a quick indication of the presence of cerebrovascular pathology (Table 2). As is widely practiced, the brain from CVD cases is cut in the coronal plane throughout. This is irrespective of whether fresh samples are dissected for freezing at autopsy or the brain is immersion fixed for later sectioning. In Newcastle, brains from CVD cases are sliced fresh in the coronal plane and then alternate sections from each hemisphere are retained as fixed or frozen material, which is deposited in the Newcastle Brain Tissue Resource. While unconventional, it has been the normal practice for the past 30 years in Newcastle to sample large sections (average size 6 × 5 cm) for better appreciation of pathology, but this is not necessarily the case in many laboratories (Fig. 2).

What is the best strategy for brain sampling? Various recommendations for brain region sampling and histological evaluation in a stratified fashion have been made (Table 2). Block sampling is recommended from the middle frontal gyrus, superior and middle temporal gyri, inferior parietal lobule and occipital cortex; in addition, the medulla, pons (including locus coeruleus), cerebellar cortex (including dentate nucleus), thalamus and subthalamic nucleus, basal ganglia at the level of the anterior commissure, hippocampus and entorhinal cortex, anterior cingulate gyrus and amygdala [81, 111] may also be considered. The BrainNet Europe Consortium has previously recommended a sampling strategy that may be adapted for instances when consent is not available to retain the whole brain for diagnostic evaluation [2]. While these are biased towards neurofibrillary pathology and involve sections from the occipital cortex, superior and middle temporal gyrus, anterior hippocampus and/or amygdala and posterior hippocampus at the level of the lateral geniculate body, they are not ideal or sufficient for scoring vascular pathology. The minimal sample set for scoring vascular pathology would include sections of the frontal lobe at the level of the olfactory bulbs, the temporal lobe at the level of the anterior hippocampus and the basal ganglia (lenticular nucleus and anterior thalamus) at the level of the mamillary body [39]. The posterior hippocampus is included if available. These regions represent relevant cerebral systems involved in cognition and receive blood from each major cerebral arterial supply [39]. The National Institute on Aging-Alzheimer’s Association (NIA-AA) recommends the assessment of hippocampal sclerosis, vascular brain injury and microvascular lesions in 12 regions [81]. However, as correctly recommended by the BrainNet Consortium [3], a simple strategy regarding assessment of load of alteration is urgently needed to yield reproducible and, at the same time, comparable results between centres.

At most centres, the histological evaluation of vascular pathology or brain tissue injury is undertaken in a rather subjective manner and is remarkably variable (Table 3). Degrees of vessel, e.g. arteriosclerosis and tissue changes (vascular brain injury) in terms of infarcts and rarefaction, often reported as a composite semi-quantitative score, are noted to validate the clinical picture. This is probably adequate for routine neuropathology reporting taking into account the nature and extent of specific changes in the vascular anatomy and the parenchyma (Table 2). More rigorous and objective analysis is time consuming and tedious and is more suitable for research purposes. However, various methods for more accurate determination have been used to quantify the vascular pathology. Table 4 provides the details of various methods which can be implemented for quantification of vascular and relevant cellular changes.

**Table 4:** Markers and quantification of vascular pathology and cells in CVD

	Pathology	Quantitative method(s)	Stain/markers	References
Blood vessels				
Arteries	Atherosclerosis	Visual grading of degree (0–3 scale) of stenosis in basal (Circle of Willis) arteries. Validated by comparison with detailed cross-sectional measurements in vessel segments	H&E	[15, 141]
	Arteriolosclerosis	Sclerotic index (SI)-ratio of outer and internal diameters; degree of loss of VSMC (0–3 scale)	H&E	[36, 99]
	Amyloid angiopathy	CAA rating: parenchymal and meningeal 0–3 scale, capillary CAA as present/absent and vasculopathy 0–2 scale	Aβ peptides; H&E	[106]
Veins	Collagenosis	Degree of wall thickening (0–3 scale)	COL4; H&E, Masson trichrome	[36]
Capillaries	Endothelium	EC degeneration, capillary length density (2D/3D stereology)	GLUT1, CD31; ICAM-1	[25, 61, 89]
	Basement membrane	Measurement of thickness of capillary wall	COL4, Laminin	[36]
Parenchymal changes	Large infarcts (3 × 3 × 2 cm)	Lesion counts 1–3	H&E	[81]
	Small infarcts/lacunes (0.5 × 0.3 × 0.2 cm)	Lesion counts >3	H&E	[39, 81, 179]
	Microinfarcts <0.2 cm	Lesion counts >3	H&E	[6, 178, 180]
	Perivascular spaces	Density (0–3); volume measurements	H&E; LFB	[186]
White matter	Myelin loss	Myelin index (MI); ratio of loss against total density. Myelin-associated glycoprotein to proteolipid protein 1 (MAG:PLP1) ratio	LFB, Loyez; MAG, PLP	[82, 163]
	Axons	Axonal (light and electron)	NF, SMI32, APP	[36]
	Oligodendrocytes	Cell counts		[82, 186]
Choroid plexus	Epithelial cells	Cell density or counts of Biondi rings inclusions	GLUT1; Thioflavin S, Tight junction proteins	[184]
Reactive Cells	Astrogliosis	Hyperplasia, hypertrophy; density by in vitro imaging	GFAP, ADH1L1	[32]
	Microglia	Reactivity, proliferation; density by in vitro imaging	CD68, Iba1	[152]
Infiltrating cells	Leukocytes, neutrophils, macrophages	Perivascular cuffing (0–3 scale), perivascular cell density in contact or within 0.5 mm circumference	CD4, Iba1, various markers	[20]
Neurons	Neuronal number	3D stereology	NeuN, SMI31	[57, 59, 188]
	Neuronal volume	3D stereology	H&E	[57, 59]
	Sclerosis (hippocampal)	HS by visual grading Type 0–4	H&E; neurodegenerative pathology antigens	[137]

Information retrieved from several references as shown. This is not an exhaustive list.

*Aβ* amyloid β, *ADH1L1* aldehyde dehydrogenase 1 family, member L1, *APP* amyloid precursor protein, *COL4* collagen IV, *GFAP* glial fibrillary acidic protein, *GLUT1* glucose transporter 1, *H&E* haematoxylin and eosin, *HS* hippocampal sclerosis, *ICAM-1* intercellular adhesion molecule-1, *LFB* luxol fast blue, *MAG* myelin associated protein, *NeuN* neuronal N protein, *PLP* proteolipoprotein

## Cerebrovascular pathology and brain parenchymal changes

Atherosclerotic and embolic disease are the main causes of infarctions associated with major arterial territories, which may be admixed in the cortical and subcortical regions [70] (Table 1). Thromboembolic events are responsible for up to 50 % of all ischaemic strokes, whereas intracranial SVD causes 25 % of the infarcts. Small vessel alternations involve arteriolosclerosis and hyalinosis and associated with lacunar infarcts predominantly occurring in the WM, basal ganglia and thalamus. WM disease or subcortical leukoencephalopathy with incomplete infarction is a common pathological change associated with dementia [39]. Other features include border zone (watershed) infarctions, laminar necrosis and cerebral amyloid angiopathy (CAA). Complicated angiopathies such as fibromuscular dysplasia, arterial dissections, granulomatous angiitis, collagen vascular disease and giant cell arteritis are rarer causes of CVD and VaD (Table 1).

Few studies have recorded precise ischaemic, oedematous and haemorrhagic lesions induced by pathological changes in the brain circulation or perfusion to be associated with VaD (Table 2). In ten different studies where VaD was diagnosed, clinically, 78 % of the cases revealed cortical and subcortical infarcts suggesting that other vascular pathologies involving incomplete infarction or border zone infarcts could be important factors. Among other lesions 25 % of the cases had cystic infarcts whereas 50 % showed lacunar infarcts or microinfarcts. Lacunar infarcts, however, appear to be a common category of infarcts and currently recognised as the most frequent cause of stroke (Table 2). Severe CAA was present in 10 % of the cases. Hippocampal sclerosis and cell atrophy, which may be caused by remote ischaemic injury, was apparent in 55 % of the cases in one study with clinical diagnosis of ischaemic VaD [173]. In an attempt to evaluate the natural history and staging of CVD, Deramecourt et al. [39] proposed that vessel wall modifications such as arteriolosclerosis or CAA were the most common and earliest changes. These were followed by perivascular spacing with lacunar and regional microinfarcts infarcts occurring as consequent, but independent processes. The regional progression of the changes were frontal > temporal lobe ≥ basal ganglia. In dementia subjects, VaD had the highest total scores of vascular pathology, whereas AD was the second and dementia with Lewy bodies was the last but greater than in ageing controls [39].

## Interaction between vascular and Alzheimer type of pathologies

**Fig. 3:**
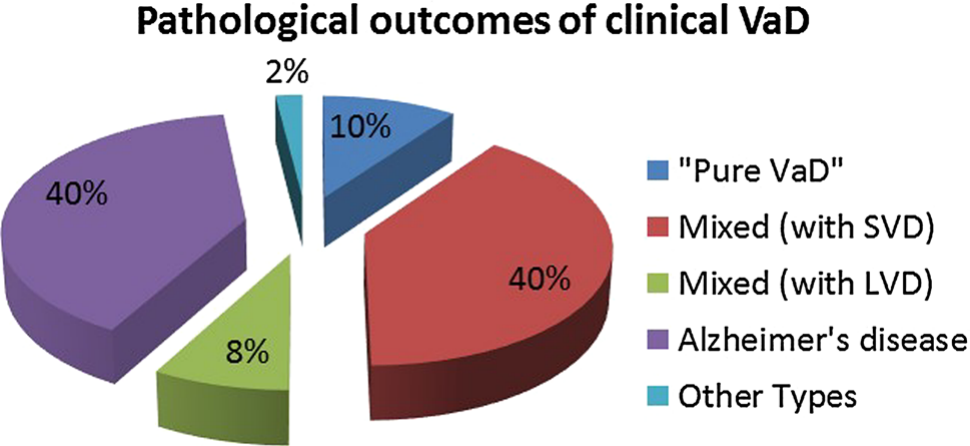
Pathological outcomes of clinically diagnosed VaD. Mixed type 1 revealed large infracts, whereas mixed type 2 predominantly exhibited SVD with microinfarction. Other included Lewy body disease, dementia, mild Parkinson disease and depression. *AD* Alzheimer’s disease

Concurrent CVD is a common neuropathological finding in aged subjects with dementia and more common in AD than in other neurodegenerative disorders, especially in younger subjects. This is evident not only in samples from memory clinics we first evaluated over 20 years ago [136], but also in those from large multicentre studies [164]. In the National Alzheimer’s Coordinating Centre minimum data set sample of 4429 clinically diagnosed AD cases, the presence of CVD and any vascular pathology was reported to be 32 and 80 % respectively. Approximately, 20 % of these had lacunes and microinfarcts [164]. The admixture of CVD pathology and neurodegenerative changes particularly neurofibrillary and α-synuclein pathologies is even greater in elderly people within the community at large [29, 140, 157]. The co-occurrence of CVD lowers the threshold for dementia caused by a single neurodegenerative process. In one community-based sample, 38 % of dementia cases had mixed pathology, with both Alzheimer-type changes and vascular lesions, but ‘pure AD’ represented only 21–24 % of the cases [148]. WM lesions indicating ischaemic or oligaemic aetiology are also high in community-dwelling subjects by as much as 94 %, and this change is an independent substrate for dementia [50]. In addition, atherosclerosis in cerebral arteries and the circle of Willis [141, 187] is frequently present in AD. The commonest overlapping pathologies involve smaller cerebrovascular lesions rather than large infarcts [39] (Fig. 3). These include most features of SVD such as cortical infarcts, lacunes, diffuse and periventricular myelin loss, WM microvacuolation, microinfarcts, microhaemorrhages, arteriolosclerosis and focal and diffuse gliosis [10, 48, 173]. AD pathology was found to be three times greater in VaD cases with small (<15 mL) compared to large infarcts [10]. The findings also corroborate the importance of microvascular disease rather than large vessel disease as the critical substrate in VaD and AD.

Clinicopathological studies also suggest that vascular disease not only influences the burden of the neurodegenerative lesion [140, 190]. The density of neocortical plaques was lower in AD cases with coexistent vascular lesions interpreted as contributing to dementia [113]. In the Religious Order study, elderly nuns who exhibited coexistent AD and brain infarcts at autopsy had poorer cognitive function and a higher prevalence of dementia than those without vascular change [156]. Compared with pure AD, the lower burden of Alzheimer-type pathology, particularly fewer neurofibrillary tangles, was required to reach the threshold for dementia when there were concomitant lacunar infarcts in subcortical structures including the basal ganglia, thalamus or deep WM. Similarly, in another religious order study, after accounting for AD lesion burden, the presence of other pathologies or infarcts increased the odds of dementia over fivefold [148] and caused earlier onset of dementia [47].

## Pathology of extra- and intracranial large vessels

Large infarction or macroinfarction should be visible upon gross examination of the brain at autopsy. Stenosis arising from atherosclerosis within large vessels is considered the main cause of large infarction, which may sometimes extend beyond the arterial territories. The stages of atherosclerosis may vary from accumulation of foam cells causing fatty streaks to complicated atheromas involving extracellular matrix components and even viral or bacterial infections [88]. Approximately, 15 % of VaD assumes occlusion of the extracranial arteries such as the internal carotid artery and the main intracranial arteries of the circle of Willis including the middle cerebral artery, leading to multiple infarcts and dementia [24]. The differences between the anterior versus posterior portions of the circle of Willis and left versus right sides may be variable, and stenosis of major arteries could be up to 75 % in very severe cases. Typical atherosclerosis or microatheromatous disease in the meningeal and smaller vessels, beyond the circle of Willis involving the proximal segments of the middle and anterior cerebral arteries, is generally rare, but may be found in very old subjects [91]. The presence of dolichoectasia and fusiform aneurysms has also been noted in some cases. In severe cases, medium-sized arteries in the leptomeninges and proximal perforating arteries are involved. The damage could be worse depending on the presence of hypertension.

Arterial territorial infarctions involve four principal areas, particularly those supplied by the major arteries: anterior, middle cerebral artery, posterior artery and the territory between the anterior and middle cerebral artery. The intensity of gliosis, both astrocytic and microgliosis, is an important consideration in judging the degree and age of infarction. However, there is no clear evidence to suggest these are related to cognitive impairment. Degrees of gliosis or glial scars are noted in brains subjected to global ischaemia, i.e. after transient cardiac arrest where responses may be observed in vulnerable neuronal groups within the hippocampus or neocortical laminae.

## Small cerebral vessels

**Fig. 4:**
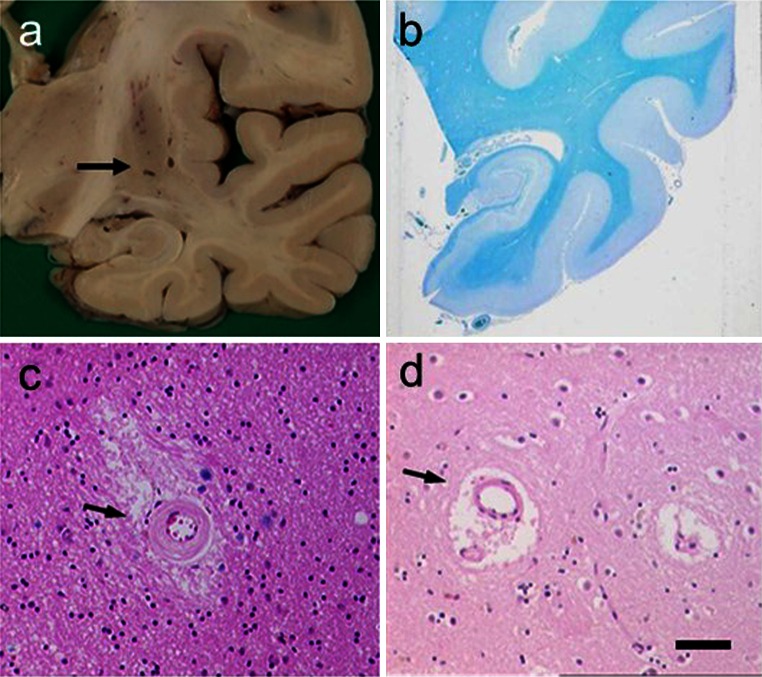
Pathological features associated with SVD in VaD. *Panels* show examples of lacunes, small infarcts and microinfarcts. **a** Typical cavitated lacunar lesions (*arrow*) in the putamen of a 65-year-old man. **b** WM attenuation in the medial temporal lobe, but sparing of U fibres. Section from an 80-year-old man with vascular and neurofibrillary pathology. **c**, **d** Cerebral microvessels with variable hyalinosis, perivascular rarefaction, microinfarcts and perivascular spaces in two different cases. Moderate gliosis in the surrounding region is also evident in the case in **c**. **d** Perivascular dilatation (or spacing) in the WM (*arrow*). *Magnification bar* **a** 1 cm, **b** 500 μm, **c**, **d** 100 μm

SVD entails fibroid necrosis, hyalinisation of vessels, expansion of the perivascular space and pallor of adjacent perivascular myelin, with associated astrocytic gliosis (Fig. 4). The smaller vessels of the brain including intracerebral end arteries and arterioles undergo progressive age-related changes [99], which alter perfusion and cause lacunar infarcts (cystic lesions generally <1 cm) and microinfarcts. The arteriolar changes range from wall thickening by hyalinosis, reduction or increment of the intima to severe arteriolosclerosis and fibroid necrosis. Arteriolosclerotic changes likely promote loss of elasticity to dilate and constrict in response to variations of systemic blood pressure or auto-regulation, which in turn causes fluctuations in blood flow response and changes in tissue perfusion. The deep cerebral structures and WM would be rendered most vulnerable, because the vessels are end arteries almost devoid of anastomoses. Small vessel pathology could also lead to oedema and damage of the blood–brain barrier (BBB) with chronic leakage of fluid and macromolecules in the WM [61, 78, 175]. Microvascular disease may also be associated with degrees of inflammation including the presence of lymphocytes or macrophages localised on blood vessels (and not necessarily a function of brain ischaemia). In the oldest SVD subjects, there may also often be evidence of remote haemorrhage in the form of perivascular hemosiderin [39].

## Lacunar infarction

Lacunar infarcts, about 1 cm or less in diameter, may occur as complete or cavitating lesions frequently in both subcortical grey and WM in VaD. They represent small foci of ischaemic necrosis resulting from narrowing or occlusion of penetrating arteries branching directly from larger cerebral arteries [56]. Lacunar infarcts are frequently multiple and bilateral and often coexist with other vascular lesions (e.g. large infarcts or diffuse WM damage). Whether single or multiple, they may be asymptomatic, depending on their location and the volume of normal brain tissue lost. Lacunes may also represent small haemorrhages or dilated perivascular spaces without infarction or haemorrhage. A few lacunes may represent healed or re-absorbed as minute or petechial haemorrhages. Microlacunes have also been described which essentially should be thought of as large cystic microinfarcts.

Apart from critical lesions occurring often in the internal capsule or caudate nucleus, recent meta-analyses suggested there were no pathological differences between symptomatic and asymptomatic patients. Perivascular oedema and thickening, and inflammation and disintegration of the arteriolar wall were common, whereas vessel occlusion was rare [9], In neuropathological studies of elderly patients with vascular disease but without evidence of AD or other neurodegenerative pathologies, dementia was associated with severe cribriform change and associated subcortical WM damage and microinfarcts [48, 158]. In the Honolulu–Asia Aging Study (HAAS) analysis [179], microvascular infarcts (lacunar and microinfarcts) were identified as the sole or dominant lesion in 34 % of the definitely impaired decedents. Only leukoencephalopathy was associated with dementia, and large infarcts were associated with VaD. VaD without significant AD pathology shows more severe cribriform change and deep white and grey matter lacunar or microinfarcts than stroke subjects with macroscopic infarcts and elderly subjects without dementia [155]. Similarly, lacunar infarcts and microinfarcts were the most common neuropathological features in more than 50 % of elderly patients with ischaemic VaD [173] and also strong determinants of dementia in the Geneva brain ageing study [60]. However, all these findings were also often accompanied by moderate to severe atherosclerosis.

## White matter changes

**Fig. 5:**
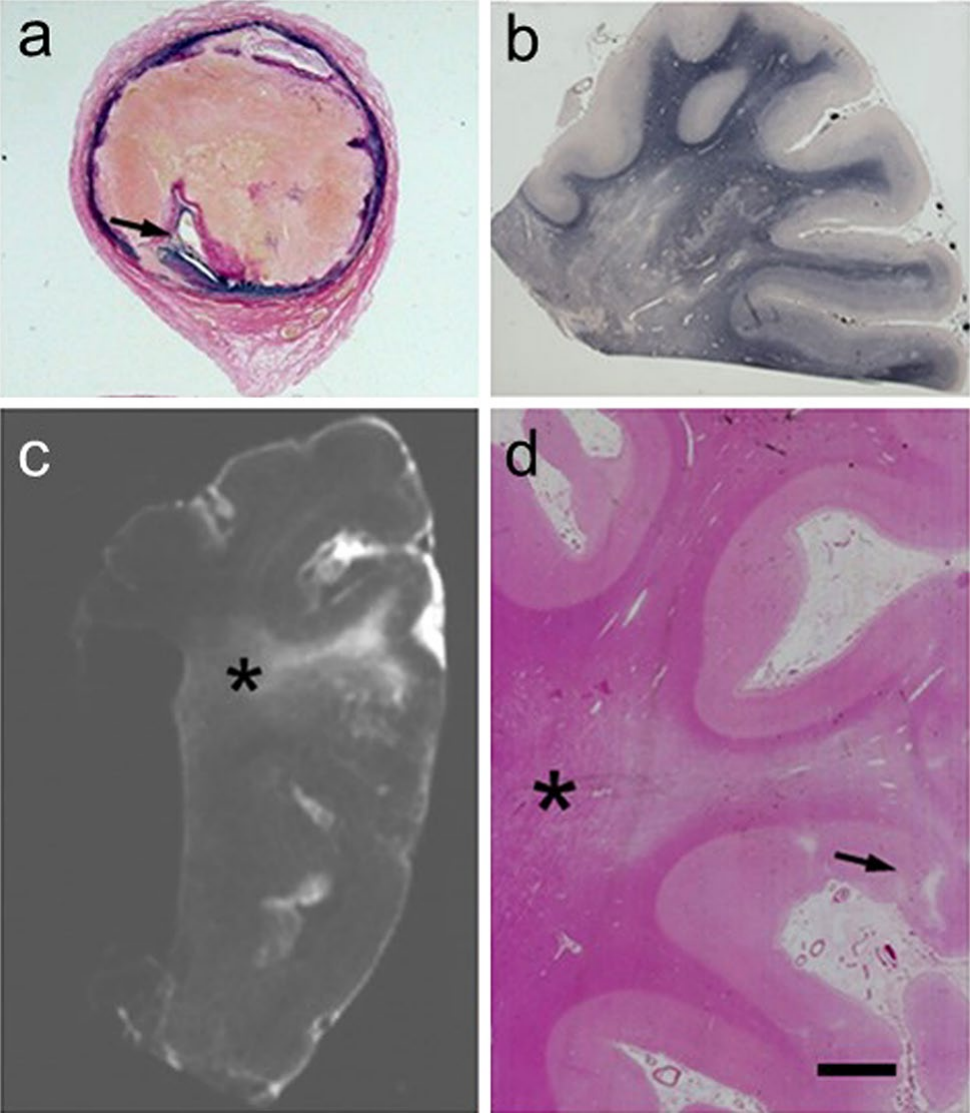
WM lesions visualised by conventional histopathological staining in a 69-year-old man diagnosed with vascular encephalopathy (and VaD). **a** >75 % stenosis in the internal carotid artery 8 mm above the bifurcation. The narrowed lumen (*arrow*) is seen. **b** Severe WM changes in the parietal lobe in this patient. Braak staging was graded as IV, but there were no neuritic or cored plaques. **c** Postmortem T2W magnetic resonance image of a formalin-fixed block from the parietal lobe. The area of hypersignal can be seen in the WM (*asterisk*). **d** H&E stained section from the block in **c** showing severe deep WM pallor in the area of hypertensity (*asterisk*). A small cortical infarct is also seen (*arrow*). *Magnification bar* **a** 500 mm, **b** 400 μm, **c** 1 cm, **d** 500 μm

White matter hyperintensities on T2-weighted MRI or leukoaraiosis as a decreased signal on computed tomography (CT) is a neuroimaging construct to describe diffuse and focal WM changes. Leukoariaosis predominantly has reference to vascular disease. It not only incorporates WM rarefaction, incomplete infarction, lacunar strokes, perivascular spacing and demyelination, but sometimes also axonal degeneration (Figs. 4 and 5). Both, areas of leukoaraiosis and zones outside the lesions show decreased vascular density indicating that leukoaraiosis appears as a generalised feature of CVD rather than being limited to the deep WM. This is consistent with the finding of an association of unstable carotid plaques with the number of WM lesions, suggesting a thromboembolic role in some patients with leukoaraiosis [5].

Neuroimaging and pathological studies demonstrate that WM hyperintensisties represent degeneration of the WM mostly explained by SVD [125, 126, 158]. Diffuse and focal WM lesions are a hallmark of VaD [82], but also occur most in ~30 % of AD and dementia with Lewy body (DLB) cases [44]. There is some controversy whether deep or periventricular lesions are of more importance, but this depends on the definition of boundaries between the periventricular and deep WM if the coursing of the fibres is used as markers [96]. Lacunar infarcts are produced when the ischaemic damage is focal and of sufficient severity to result in a small area of necrosis, whereas diffuse WM change is considered a form of rarefaction or incomplete infarction where there may be selective damage to some cellular components. Although the U fibres are frequently spared WM disease may comprise several patterns of alterations including pallor or swelling of myelin, loss of oligodendrocytes, damage to axons, cavitations with or without the presence of macrophages and areas of reactive astrogliosis [152], where the astrocytic cytoplasm and cell processes may be visible with standard stains. Oligodendrocytes are particularly vulnerable to hypoxic environment created by low perfusion, which in turn may differentially affect myelin as indicated by the remarkable reduction in the ratio of myelin-associated glycoprotein (MAG) to proteolipid protein 1 (PLP1) not only in the WM, but also the cerebral cortex in VaD [12, 163].

Lesions in the WM also include spongiosis, i.e. vacuolisation of the WM structures and widening of the perivascular spaces [186]. The affected regions do not have sharp boundaries, in contrast to the plaques of multiple sclerosis. These changes may be associated with chronic pro-thrombotic endothelial dysfunction in cerebral SVD [77] also involving the WM [23]. There may be a cerebral response to the SVD by increasing endothelial thrombomodulin [61]. The projected misery perfusion due to capillary loss or abnormalities occurring prior to leukoaraiosis corroborates the finding of a chronic hypoxic state in the deep WM [51], which also releases several growth promoting factors [153]. Some of the WM damage in demented patients may simply reflect Wallerian changes secondary to cortical loss of neurons. However, histological changes characteristic of Wallerian degeneration are not readily evident as WM pallor. Conversely, in AD patients with severe loss of cortical neurons, similar WM lesions are not apparent [44].

While WM changes focus on the arterial system, narrowing and, in many cases, occlusion of veins and venules by collagenous thickening of the vessel walls also occur. The thickening of the walls of periventricular veins and venules by collagen (collagenosis) increases with age, and perivenous collagenosis is increased further in brains with leukoaraiosis [22]. The presence of apoptotic cells in WM adjacent to areas of leukoaraiosis suggests that such lesions are dynamic, with progressive cell loss and expansion [22]. Vascular stenosis caused by collagenosis may induce chronic ischaemia or oedema in the deep WM leading to capillary loss and more widespread effects on the brain [23].

## Cerebral microinfarction

The accumulation of small, even miniscule ischaemic lesions as an important substrate of VaD has been emphasised in recent years [86]. Microinfarcts are widely accepted to be small lesions visible only upon microscopy (Fig. 4; Table 3). These lesions of up to 5 mm diameter may or may not involve a small vessel at its centre, but are foci with pallor, neuronal loss, axonal damage (WM) and gliosis. They are estimated to occur in thousands [178]. Sometimes these may include regions of incomplete infarction or rarefied (subacute) change. Microinfarcts have been described as attenuated lesions of indistinct nature occurring in both cortical and subcortical regions. Such lesions or a combination of these are reported when there are multiple or at least greater than three present in any region (Table 2). Microvascular infarcts (lacunar infarcts and microinfarcts) appear central to the most common cause of VaD (Fig. 4) and predict poor outcome in the elderly [10, 21, 173]. Interestingly, in the autopsied older HAAS, the importance of microvascular lesions as a likely explanation for dementia was nearly equal to that of Alzheimer lesions [179, 180]. Microinfarction in the subcortical structures has been emphasised as a substrate of cognitive impairment [6, 86] and correlated with increased Alzheimer type of pathology, but cortical microinfarcts also appear to contribute to the progression of cognitive deficits in brain ageing [97].

**Fig. 6:**
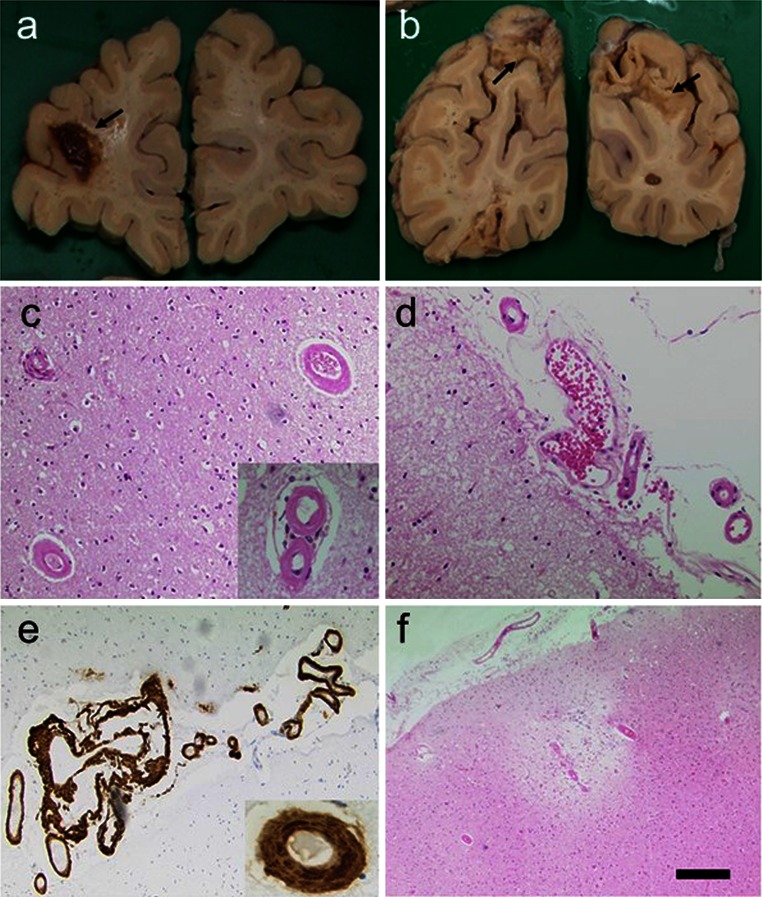
CAA and infarcts in a 92-year-old woman with memory loss, confusional state and disorientation. CT on admission showed infarction in the *right* posterior parieto-occipital region. Small lacunar infarct in the posterior aspect of the *left* corona radiata, probable area of cortical infarction in the *left* occipital lobe. **a** Lobar haemorrhage in the frontal lobe. **b** Macroscopical cortical infarcts in both *right* and *left* occipital lobes. **c**, **d** Cortical and subarachnoidal arterioles showing thickened homogenous eosinophilic walls. *Inset* in **c**, two strongly stained eosinophilic vessels. **e** Aβ immunohistochemistry shows extensive subarachnoidal and cortical amyloid angiopathy. **f** A cortical microinfarct with haemosiderin. There were numerous microinfarcts in the frontal, parietal and occipital cortices. Subject only showed sparse cored and diffuse senile plaques and Braak stage II for neurofibrillary pathology. *Magnification bar* **a**, **b** 1 cm, **c**–**f** 100 μm

In addition to microinfarction in the subcortical structures, it appears increasingly important that multiple cortical areas of microinfarction are associated with subcortical VaD or SVD (Fig. 4). Thus, these lesions should be taken into account when defining the neuropathological criteria. Cortical microinfarcts are increased in the presence of CAA [121] (Fig. 6). In a recent study, cortical microinfarcts were frequently detected in AD and associated with CAA, but rarely observed in subcortical VaD linked to SVD [120, 159]. Microinfarcts in the cerebral cortex associated with severe CAA may be the primary pathological substrate in a significant proportion of VaD cases [74]. Cortical microinfarcts and to lesser extent periventricular demyelination were significantly associated with cognitive decline in individuals at high risk for dementia [63]. It is proposed the changes in hemodynamics, e.g. hypotension and atherosclerosis may play a role in the genesis of cortical watershed microinfarcts.

## Large and small cerebral haemorrhages

Cerebral microbleeds detected by MRI are small, dot-like hypotense abnormalities, have been associated with extravasated haemosiderin derived from erythrocytes, lipohyalinosis and CAA [49]. They are likely a surrogate marker of SVD evident on MRI along with lacunes and WM changes [168]. The prevalence of radiological microbleeds in VaD ranges 35–85 %. Microbleeds are mainly thought to result from hypertensive vasculopathy, but the frequent co-occurrence of lobar microbleeds suggests that the neurodegenerative pathology or CAA is also of importance [177]. The relevance of this radiological construct is increasingly recognised due to their relation to clinical outcome and occurrence in anti-amyloid vaccination trials [69]. However, the presence of multiple microbleeds in the context of VaD is related to worse performance on cognitive tests, mainly in psychomotor speed and executive functioning. Since microbleeds are common in cognitively normal older individuals, attribution of these to VaD should follow a careful exclusion of other causes of cognitive impairment and only if numerous such lesions are present.

Both radiological cerebral microbleeds and foci of haemosiderin containing single crystalloids or larger perivascular aggregates are found in brains of older subjects including those diagnosed with VaD and AD, but the radiological and pathological relationship between these findings has not been entirely clear. Recent evidence suggests that cerebral microbleeds detected by MR imaging are a surrogate for ischaemic SVD rather than exclusively haemorrhagic diathesis [83]. Greater putamen haemosiderin was significantly associated with indices of small vessel ischaemia, including microinfarcts, arteriolosclerosis and perivascular spacing and with lacunes in any brain region but not large vessel disease, or whole brain measures of neurodegenerative pathology. Higher levels of putamen haemosiderin were correlated with more microbleeds upon MR imaging, but it is possible that brain iron homoeostasis and small vessel ischaemic change are responsible for these rather than only as a marker for minor episodes of cerebrovascular extravasation.

## Hippocampal atrophy and sclerosis

Neuroimaging studies have shown that medial temporal lobe and hippocampal atrophy are associated with VaD [14, 54] and SVD [119, 167], albeit not to the same extent as in AD [26]. Pathological evidence shows that ischaemic VaD and SVD are also associated with hippocampal changes and atrophy remote to ischaemic injury [108, 188]. Hippocampal neurones in the Sommer’s sector are highly vulnerable to disturbances in the cerebral circulation or hypoxia caused by systemic vascular disease. The focal loss of CA1 neurons in ischemic VaD has been related to lower hippocampal volume and memory score [188], but the degree of loss appears less in VaD [98] than in AD. However, selective hippocampal neuronal shrinkage is also an important substrate for VaD. This is also evident in delayed dementia after stroke in the absence of any neurodegenerative pathology [59]. Thus, there is a clear vascular basis for hippocampal neurodegeneration and concurs with the neuroimaging observations of hippocampal atrophy even in population-based incident VaD [147]. The simplest mechanistic explanation for the atrophy is that the neuronal or dendritic arbour results in subsequent loss in connectivity, which contributes to brain structural and functional changes. This is consistent with the finding that soluble synaptophysin was decreased in VaD as well as AD.

Hippocampal sclerosis is a likely a major contributing factor in the hippocampal atrophy and occurs in approximately 10 % of individuals over the age of 85 years and slightly higher in VaD. It is characterised by severe cell loss with the CA fields in the presence or absence of microinfarction and gliosis that is not explained by AD. TAR DNA protein 43 immunohistochemistry can be used to demonstrate that hippocampal sclerosis, regardless of accompanying pathologies (e.g. AD or vascular), is consistent with an underlying neurodegenerative pathogenetic mechanism [189]. Any focal loss or patterns of hippocampal sclerosis can be graded [137] and recorded together with any microinfarctions. Sometimes, the patchy neuron loss and gliosis in some brains with AD pathology may be difficult or impossible to distinguish from anoxic–ischemic change. The aetiology of hippocampal sclerosis is defined in association with a neurodegenerative process, a pathologic condition presumed to arise from hypoxic/ischemic mechanisms [137]. Hippocampal sclerosis pathology can be associated with different underlying causes, but a large study [115] found no evidence for associations between hippocampal sclerosis and lacunar infarcts, large infarcts, Circle of Willis atherosclerosis or CAA. However, there was a correlation between hippocampal sclerosis and arteriolosclerosis in multiple brain regions outside of the hippocampus including the frontal cortex (Brodmann area 9) [115]. This is ascribed to a pathogenetic change in aged human brain arterioles that impacts multiple brain areas and contributes to hippocampal sclerosis of ageing [116].

## Border zone and watershed infarcts

The border zone or watershed infarctions mostly occur from haemodynamic events, usually in patients with severe internal carotid artery stenosis. They could occur bilaterally or unilaterally and disposed to regions between two main arterial territories, deep and superficial vessel systems. Typical border zone infarctions may be 5 mm or more wide as wedge-shaped regions of pallor and rarefaction extending into the WM. Larger areas of incomplete infarction may extend into the WM [82]. These are characterised by mild to moderate loss of oligodendrocytes, myelin and axons in areas where there may be hyalinised vessels [24]. This may be accompanied by astrogliosis, some microgliosis and macrophage infiltration. The morphology of incomplete or subinfarctive changes, though suspected to be associated with cognitive function, is not consistently described in VaD. It may variably manifest as tissue rarefaction assessed by conventional stains and revealed as injury response such as microgliosis and astrocytosis, or the presence of other “reactive” cells or surrogate markers of dendritic, synaptic or axonal damage.

## Laminar necrosis

Laminar necrosis is characterised by neuronal ischaemic changes leading to neuronal loss and gliosis in the cortical ribbon. This is particularly apparent in cases where global ischaemia or hypoperfusion has occurred as in cardiac arrest. Typical topographic distribution of spongiform change can be readily apparent with standard stains. They appear more commonly at the arterial border zones [24, 88] that may fall into the subtype IV of VaD pathology.

## Sporadic cerebral amyloid angiopathy in VaD

CAA occurs most commonly in AD [8, 31, 70, 106], but it often occurs in CVD in the absence of Alzheimer pathology [34]. CAA is an independent substrate for cognitive impairment and contributes to cognitive dysfunction [7, 131]. Tissue microstructural damage caused by CAA prior to pre-intracerebral haemorrhage is also independently associated with cognitive impairment [174]. The prevalence of CAA in VaD is not known, but it is a major cause of intracerebral and lobar haemorrhages leading to profound ischaemic damage [138] (Fig. 6). Several familial forms of CAA involving ischaemic and haemorrhagic infarcts (see below) and cerebral hypoperfusion demonstrate the link between CAA and VaD. In a study of surgical biopsies exhibiting cerebral and cerebellar infarction, CAA was significantly more common in samples showing infarction than in age-matched controls with non-vascular lesions [27]. There is also an association between severe CAA and cerebrovascular lesions coexisting with AD, including lacunar infarcts, microinfarcts and haemorrhages [43, 121, 122]. This association apparently is not attributable to apolipoprotein E (*APOE*) ε4 allele, as the vascular lesions correlated best with severity of CAA, regardless of genotype [70, 123]. There is also some evidence to suggest that CAA is related to WM changes, but not exclusively in the oldest old [160]. The first stroke-like episode triggers multiple cerebral bleeds, which is preceded by diffuse WM changes that in turn lead to rapid decline of cognitive functions.

## Neurochemical pathology of VaD

The neurochemical basis of cognitive decline in CVD is poorly understood. There are few concerted studies on the protein and lipid chemistry of VaD. Various cellular signalling and regulatory mechanisms including apoptosis, autophagy, oxidative stress and inflammation are associated with VaD by virtue of their involvement in cerebral ischaemia or oligaemia. VaD subjects also mount a selectively attenuated neuroinflammatory response [112]. It was reported that the monocyte chemoattractant protein-1 and interleukin-6 concentrations were significantly reduced in the frontal lobe of VaD and mixed dementia subjects, suggesting that these changes had a vascular basis rather than due to Alzheimer pathology.

The perivascular nerve plexus [75] is highly vulnerable, yet only few transmitter-specific changes reflecting neurovascular pathology have been described in subtypes of VaD. Selective transmitter specific changes have been described in some cases of VaD [87]. Two different groups had previously shown that compared to AD patients, choline acetyltransferase activity was also reduced albeit to a lesser degree in the temporal cortex and hippocampus in patients diagnosed with multi-infarct dementia or VaD [66, 130]. Furthermore, choline acetyltransferase activities were significantly reduced by 60–70 % in the frontal and temporal cortices of subjects with CADASIL, which models SVD [92]. Choline acetyltransferase and P75 (neurotrophin receptor) immunoreactivities were also affected within the cholinergic cell bodies of the basal forebrain in CADASIL, but these were not so pronounced. This may depend on the severity of the WM degeneration [92, 107]. However, loss of cholinergic function is consistently greater in VaD patients with concurrent Alzheimer pathology [143]. Conversely, a novel increase in cholinergic activity in the frontal cortex was revealed in infarct dementia [151].

Other studies have reported deficits in monoamines including dopamine and 5-hydroxytryptamine (5HT) in the basal ganglia and neocortex in VaD [66]. To compensate for the loss [42], 5-HT(1A) and 5-HT(2A) receptors are likely increased in the temporal cortex in multi-infarct, but not subcortical VaD. Such findings, albeit fragmentary, reveal distinctions between the neurochemical pathology of VaD subtypes and suggest possibilities of pharmacological manipulation with novel therapies in VaD. There was also loss of glutamatergic synapses, assessed by vesicular glutamate transporter 1 concentrations, in the temporal cortex of VaD [93], but preservation of these in the frontal lobe suggests a role in sustaining cognition and protecting against dementia following a stroke. However, a recent study has shown that the presynaptic synaptic proteins synaptophysin and SNAP-25 are reduced, whereas drebrin is increased possibly due to decreased synaptic input in VaD [154]. Identification of the morphological equivalents of these changes in types of pyramidal cells in the frontal lobe would be relevant.

## Pathological investigation of hereditary CAA

There are more than ten different hereditary CAAs caused by mutations in different genes [139, 185]. All these angiopathies lead to some degree of cognitive impairment or dementia. They are characterised by multiple haemorrhages and haemorrhagic or ischaemic infarcts in addition to severe amyloid deposition within walls of the meningeal and intracerebral vessels. In hereditary cerebral haemorrhage with amyloidosis of the Dutch type, dementia occurs in most patients surviving their initial stroke [71] and may occasionally be the presenting feature [176]. The extensive CAA is alone sufficient to cause dementia and this has implications for CAA-related cognitive dysfunction in sporadic CAA and AD [114]. In the Icelandic type of hereditary cerebral haemorrhage with amyloidosis (HCHWA-I), which is associated with a point mutation in the gene encoding the cysteine protease inhibitor Cystatin C [101], dementia, occurring in some patients, has been attributed to the multiple vascular lesions. Individuals with gelsolin-related amyloidosis manifest facial palsy, mild peripheral neuropathy and corneal lattice dystrophy; atrophic bulbar palsy, gait ataxia and mild cognitive impairment [94]. Familial British dementia with severe CAA [170] is characterised by dementia, progressive spastic tetraparesis and cerebellar ataxia, the onset of which is usually in the sixth decade [109]. Neuropathological features also include Alzheimer-type neurofibrillary tangles and neuropil threads in the anteromedial temporal lobe that may contribute of dementia [132, 139]. Familial Danish dementia (FDD), also known as heredopathia ophthalmo-oto-encephalica, is another condition with severe and widespread CAA [171]. FDD is characterised clinically by cataracts, deafness, progressive ataxia and dementia.

## Investigation of familial small vessel diseases

Several familial stroke disorders also appear to cause cognitive impairment or dementia. These can be diagnosed in biopsy tissues using immunohistochemical or electron microscopy methods (Table 5). A common feature in these is subcortical SVD, often characterised by severe arteriolosclerosis in the perforating vessels [185]. Cerebral autosomal dominant arteriopathy with subcortical infarcts and leukoencephalopathy (CADASIL) is the most common form of hereditary SVDs [30]. Motor deficits, an ataxic hemiparesis, hemianopsia and dysarthria may present as key neurological features akin to SVD. Vascular changes including apoptotic loss of brain vascular smooth muscle cells [68] and vessel wall thickening [37] likely reduce blood flow and affect the vasodilatory response to cause lacunar infarcts and microinfarcts in grey matter and WM [185]. The extensive demyelination and axonal damage in the underlying WM contributes to cortical atrophy [37] and impacting on frontal lobe cognitive functions that is consistent with the disconnection of the fronto-striatal circuits in CADASIL. Neuronal apoptosis, predominantly in neocortical layers III and V, also likely contributes to dementia in CADASIL [68].

**Table 5:** Sampling of tissue and fluids for diagnosis of uncommon causes of CVD

Diagnostic sample	Diagnostic test	Target diagnoses
Tissue biopsy	Arterial	Temporal or giant cell arteritis, Sneddon syndrome
	Cerebral meninges	Primary cerebral vasculitides
	Muscle	CADASIL, mitochondrial diseases
	Skin	CADASIL, CARASIL, Sneddon syndrome, psuedoxanthoma elasticum and unexplained skin lesions with CNS manifestations
CSF	GLA activity	Fabry disease
	Inflammatory cells	Vasculitides, sarcoidosis, CNS infections
Serum	Viral, bacterial	Specific infections
	ANA, ENA and anti DNA antibodies, complement	Systemic lupus erythematosus, connective tissue diseases
	Protein electrophoresis	Paraproteinaemia
	Hb electrophoresis	Sickle cell anaemia (HbS), thalassaemia
Serum and urine	Toxicology	Illicit drug use
	Ammonium	Glutaric acidaemia type 1 and 2, urea cycle disorders
	Lactic acidosis	Branch-chained organic acidurias, glutaric acidaemia type 1 and 2, mitochondrial diseases
	Thrombophilia	Antithrombin, protein C, protein S, antiphospholipid antibodies (lupus anticoagulant, anticardiolipin, β2-glycoprotein), homocysteine, factor V Leiden, prothrombin, MTHRF, factor XII

Data adapted from several references [88, 161]. Other rare conditions with stroke injury such as syphilis, systemic vasculitides and rheumatic diseases may also be diagnosed from CSF

*ANA* antinuclear antibodies, *ENA* extractable nuclear antigens, *CADASIL* cerebral autosomal dominant arteriopathy with subcortical infarcts and leukoencephalopathy, *CARASIL* cerebral autosomal recessive arteriopathy with subcortical infarcts and leukoencephalopathy, *CNS* central nervous system, *CSF* cerebrospinal fluid, *GLA* galactosidase alpha, *Hb* haemoglobin, *MTHRF* methylenetetrahydrofolate reductase

The Maeda syndrome or cerebral autosomal recessive arteriopathy with subcortical infarcts and leukoencephalopathy (CARASIL) is an autosomal recessive disorder similar to CADASIL [76]. The normotensive affected subjects exhibit not only severe arteriolosclerosis, leukoencephalopathy and lacunar infarcts, but also spinal anomalies and alopecia. Strokes lead to stepwise deterioration with most subjects becoming demented in older age. Familial cerebral SVDs involving progressive visual impairment [185] cause deterioration in cognitive function. Hereditary endotheliopathy with retinopathy, nephropathy and stroke (HERNS), cerebroretinal vasculopathy (CRV) and hereditary vascular retinopathy (HVR) were reported independently, but represent different phenotypes in the same disease spectrum [85, 124, 162]. These, now described as autosomal dominant retinal vasculopathy with cerebral leukodystrophy, lead to early death and cause dementia. Retinal microvessels undergo severe distortions and become tortuous, predictive of the SVD type of pathology with multilaminated vascular basement membranes in the brain [85].

Some rarer and less characterised hereditary SVDs (Table 1) also exist that are associated with clinical SVD features and different degrees of cognitive impairment, but the pathologies in these are not described. They include conditions with abnormalities in the skin and eye and multiple lacunar infarcts in the deep WM and pons [128], retinal arteriolar tortuosity and leukoencephalopathy [67, 166] and profound WM changes upon MRI [169].

## Summary

Defining the neuropathological substrates of VaD relies on uniformity in sampling and careful pathological examination. VaD resulting from severe VCI or vascular cognitive disorder or from delayed impairment after stroke appears to result from the accumulation of several lesions including cerebral atrophy. While robust neuropathological criteria for VaD are still being developed, multiple microinfarcts, small infarcts or lacunes in the subcortical structures rather than macroinfarction or large vessel disease appear most robustly related to cognitive impairment. Diffuse WM changes involving periventricular and deeper regions are frequent in VaD. Concomitant hippocampal pathology including sclerosis and Alzheimer pathology compound disease progression. Further definitions of the neuropathological correlates of VaD and investigation of genetic models would be valuable for exploring the pathogenesis as well as management of VaD through preventative and treatment strategies.
